# Spontaneous Lateral Sphenoid Sinus Meningocele Presenting With Recurrent Meningitis: Surgical Resolution via an Endoscopic Endonasal Transpterygoid Approach

**DOI:** 10.7759/cureus.89962

**Published:** 2025-08-13

**Authors:** Shingo Umemoto, Takayuki Matsunaga, Yasutomo Momii, Minoru Fujiki, Takashi Hirano

**Affiliations:** 1 Department of Otorhinolaryngology - Head and Neck Surgery, Faculty of Medicine, Oita University, Yufu, JPN; 2 Department of Neurosurgery, Faculty of Medicine, Oita University, Yufu, JPN

**Keywords:** cerebrospinal fluid leak, endoscopic endonasal transpterygoid approach (eetpa), meningocele, skull base reconstruction, sphenopalatine artery

## Abstract

A 52-year-old woman with a history of recurrent bacterial meningitis and clear nasal discharge was diagnosed with a spontaneous meningocele of the lateral recess of the left sphenoid sinus. Radiological evaluation revealed a bony defect lateral to the vidian rotundum line, consistent with excessive lateral pneumatization and suggestive of a skull base defect. The patient underwent endoscopic endonasal transpterygoid repair, which provided sufficient access to the lateral recess. During surgery, the sphenopalatine artery was transected to achieve full exposure, which precluded the use of an ipsilateral vascularized flap. A multilayer closure was performed by using autologous fascia lata, fat, polyglycolic acid felt (Neoveil®, GUNZE Ltd., Osaka, Japan), and fibrin glue. The surgery was performed as a collaboration between otolaryngologists and neurosurgeons. The postoperative course was uneventful, with neither cerebrospinal fluid (CSF) rhinorrhea nor meningitis recurring during five years of follow-up. This case illustrates the importance of precise anatomical assessment and individualized reconstruction strategies in the endoscopic management of lateral sphenoid sinus meningoceles.

## Introduction

Spontaneous meningocele of the lateral recess of the sphenoid sinus is a rare but clinically important condition that may lead to cerebrospinal fluid (CSF) rhinorrhea [1] or recurrent meningitis [2]. These lesions are often associated with excessive lateral pneumatization of the sphenoid sinus [3] and with bony defects, such as the persistence of Sternberg’s canal [4-7]. Recent advances in endoscopic skull base surgery have identified the endoscopic endonasal transpterygoid approach (EETPA) as the preferred route for accessing lesions beyond the vidian rotundum (VR) line, especially those involving the lateral recess [1,8,9].

Diagnosis can be challenging, particularly in cases lacking overt CSF leakage or those complicated by previous sinus surgeries. High-resolution computed tomography (HRCT), T2-weighted magnetic resonance imaging (MRI), and β-2 transferrin testing are essential for accurate localization and confirmation of the diagnosis [1]. Although multiple reports have demonstrated the effectiveness of vascularized flaps in reconstructing skull base defects, compromised flap viability from surgical interventions such as sphenopalatine artery (SPA) ligation may preclude their use [9,10].

Herein, we report the case of a woman with a lateral sphenoid sinus meningocele successfully treated with EETPA. We discuss the anatomical and surgical considerations, including reconstructive strategy in the context of SPA manipulation.

## Case presentation

History of present illness

A 52-year-old woman presented with a history of recurrent bacterial meningitis and persistent left unilateral serous rhinorrhea over the previous year. She was otherwise in good general condition at presentation. She had been admitted to a previous hospital multiple times for treatment of bacterial meningitis, with CSF cultures yielding *Streptococcus salivarius*. This was initially suspected to be secondary to sphenoid sinusitis. Consequently, she underwent endoscopic sphenoid sinus surgery at the referring hospital. However, intraoperatively, only edematous mucosa and serous fluid were observed within the sphenoid sinus; no purulent material was identified. The patient denied any history of head trauma or falls.

Although her headache, altered consciousness, and other symptoms of meningitis improved following antibacterial therapy, serous rhinorrhea persisted throughout the clinical course and did not resolve following surgery.

Diagnostic findings and treatment planning

**Figure 1:**
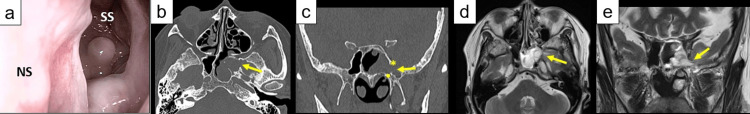
Preoperative findings. (a) Nasal endoscopy showing that the left sphenoid sinus had been opened during previous surgery. Pulsation of the lateral wall and serous fluid leakage were present. (b) Axial and (c) coronal HRCT sections showing the bony defect at the lateral fossa of the SS. (d) Axial and (e) coronal MRI (T2W1) sections showing the meningocele erupting into the SS. The arrows on panels (b-d) indicate the site of the bony defect, with the meningocele erupting through the defect. The asterisk and the dot on panel (c) indicate the foramen rotundum and vidian canal, respectively. NS, nasal septum; SS, sphenoid sinus

Given the concern for CSF rhinorrhea, the patient was referred to our hospital for further evaluation. Nasal endoscopy revealed a pulsatile area along the lateral wall of the sphenoid sinus, with continuous serous fluid leakage (Figure 1a). HRCT demonstrated extensive lateral pneumatization of the left sphenoid sinus and a bony defect in the lateral recess (Figures 1b, 1c). T2-weighted MRI revealed a high-intensity signal consistent with a meningocele (Figures 1d, 1e). The combination of continuous leakage on endoscopic nasal examination, imaging findings, and a positive β-2 transferrin assay confirmed the diagnosis of CSF leak.

Based on these findings, the patient was diagnosed with a meningocele protruding from the lateral recess of the left sphenoid sinus, complicated by CSF leakage and recurrent meningitis. Therefore, a surgical plan was made to perform endoscopic endonasal resection of the meningocele and skull base reconstruction.

Surgical findings

**Figure 2:**
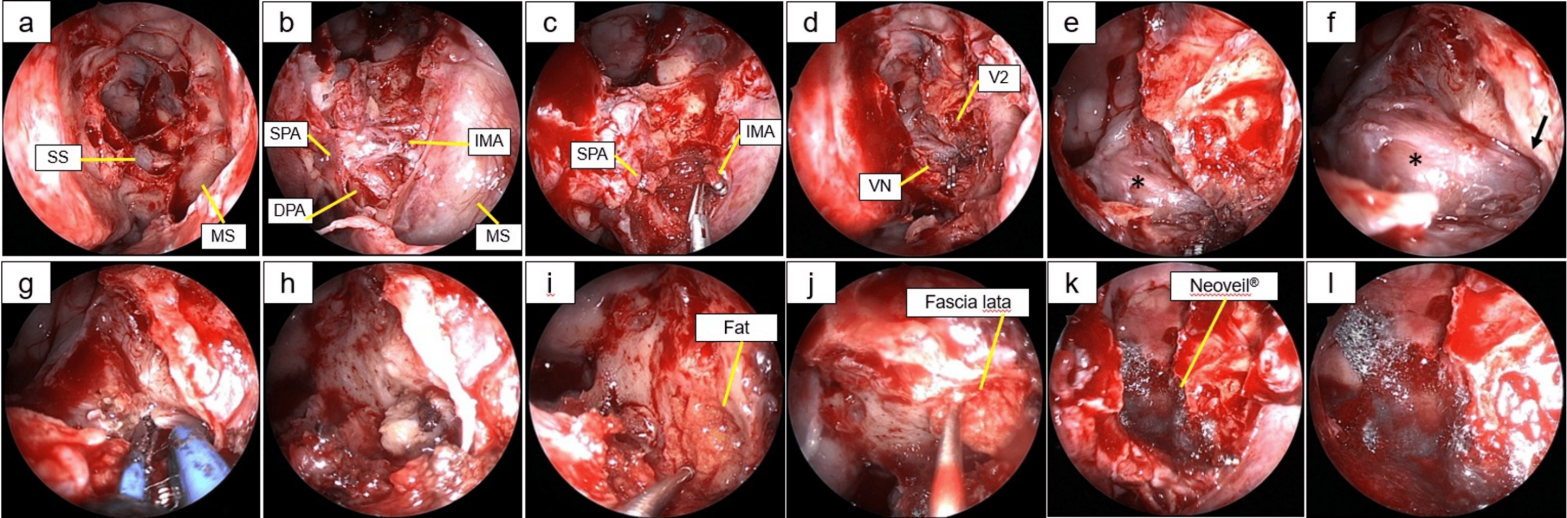
Operative course and findings during EETPA. Multi-angle endoscopic views demonstrating the localization and dissection of the meningocele, followed by multilayered reconstruction. (a) All sinuses were opened. (b) The pterygopalatine fossa was opened, and the SPA, DPA, and IMA were identified. (c) The SPA was dissected to access the lateral fossa of the SS. (d) V2 and VN were identified lateral and medial to the lateral fossa of the SS. (e) The lateral fossa of the SS was opened. (f) The bony defect was identified, with the meningeal mass erupting through it (arrow). (g, h) The meningocele was resected using an electrocautery unit. (i-l) The cranial base was reconstructed with fascia and reinforced with Neoveil® (an absorbable felt made of polyglycolic acid). The asterisks (*) on panels (e) and (f) indicate the meningocele erupting into the SS. The arrow on panel (f) indicates the bony defect in the lateral fossa of the SS. DPA, descending palatine artery; EETPA, endoscopic endonasal transpterygoid approach; IMA, internal maxillary artery; MS, maxillary sinus; SPA, sphenopalatine artery; SS, sphenoid sinus; V2, maxillary nerve; VN, vidian nerve

The patient underwent an EETPA under general anesthesia. The surgery was performed in collaboration with otolaryngologists and neurosurgeons. First, all the left sinuses were opened (Figure 2a). Next, the lateral recess of the sphenoid sinus was accessed via the pterygopalatine fossa. After removing the bony wall on the posterior aspect of the maxillary sinus to expose the pterygopalatine fossa, the soft tissue within the fossa was carefully dissected to identify the SPA. The SPA was then transected at its origin from the internal maxillary artery to improve visualization (Figures 2b, 2c). Similarly, the remaining soft tissue in the pterygopalatine fossa was dissected medially and superiorly to expose the vidian nerve (VN) and the maxillary nerve (V2), respectively. The bony wall between these two structures - the anterior wall of the lateral recess - was drilled away, allowing direct visualization of the defect. A sizable bony defect lateral to the VR line was then identified (Figures 2d-2f), and the herniated arachnoid and brain tissue were carefully resected by using an electrocautery unit (Figures 2g, 2h). Opening of the sinus cavity and accessing the pterygopalatine fossa to resect the meningocele were performed by the otolaryngologists (Figures 2a-2h). In this case, transection of the SPA was unavoidable to secure adequate exposure of the laterally and posteriorly located defect; SPA preservation was considered preoperatively but deemed technically infeasible intraoperatively.

The neurosurgeons then performed a multilayered cranial base reconstruction (Figures 2i-2l) by using thigh-derived fat (Figure 2i), autologous fascia lata (Figure 2j), and polyglycolic acid felt (Neoveil®, GUNZE Ltd., Osaka, Japan) secured with fibrin glue (Figures 2k, 2l). Because of the transection of the SPA, an ipsilateral nasoseptal flap (NSF) could not be used; however, watertight closure was achieved without it, and using a contralateral NSF was not considered necessary. Therefore, a lumbar drain (LD) was not placed.

Histopathological findings

**Figure 3:**
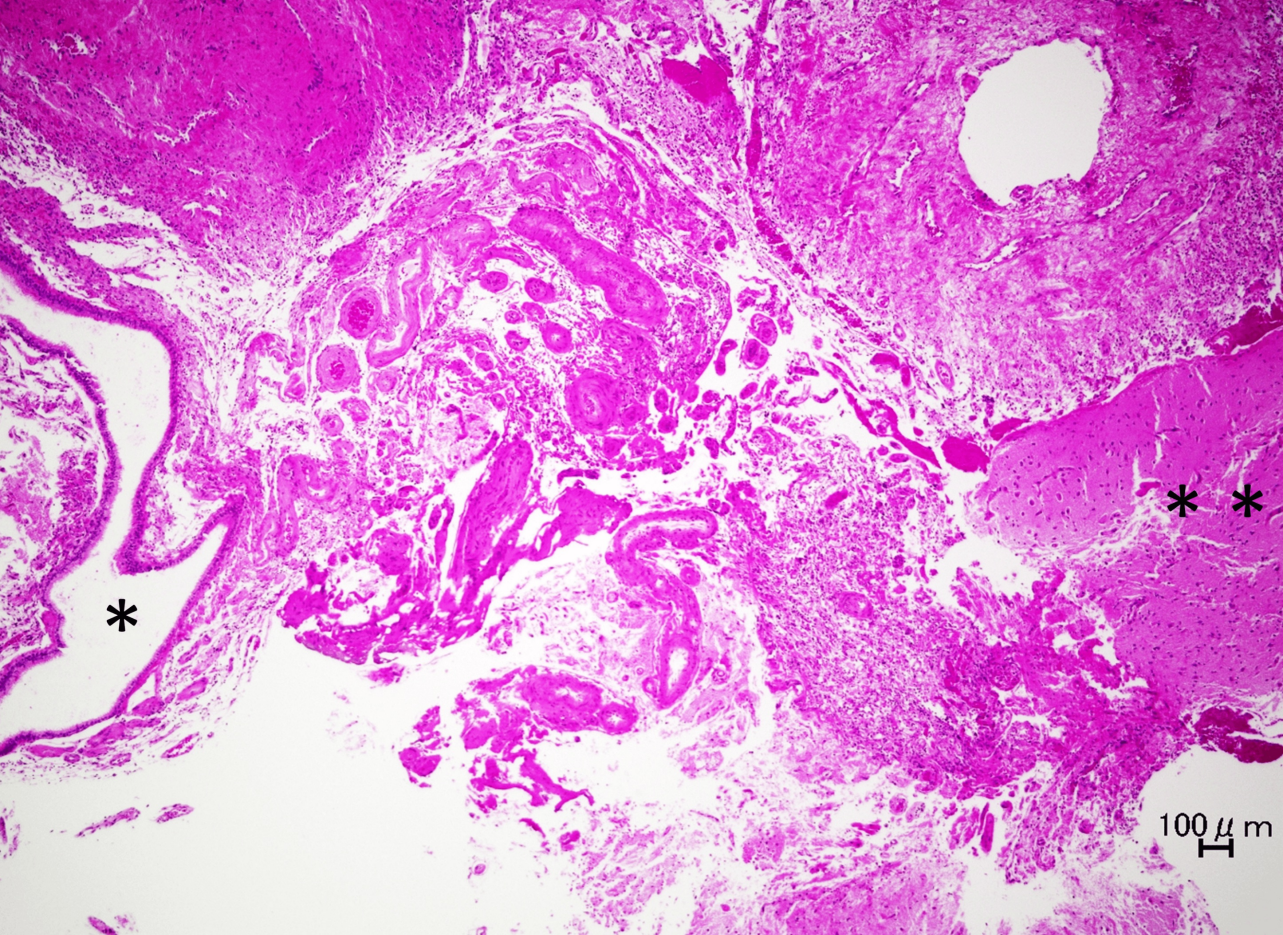
Histopathology of the resected specimen. Non-atypical multilineage epithelium consistent with sinus tissue (*) is in contact with stroma containing a spindle-shaped group of cells, suggesting central nervous tissue (**). These findings are consistent with a meningocele. Scale bar: 100 μm.

Postoperative histopathological examination of the resected tissue revealed ciliated pseudostratified epithelium, consistent with sinonasal mucosa. It was contiguous with stromal tissue with spindle-shaped cellular components, suggesting a central nervous system origin, confirming the diagnosis of a meningocele (Figure 3).

Postoperative course

**Figure 4:**
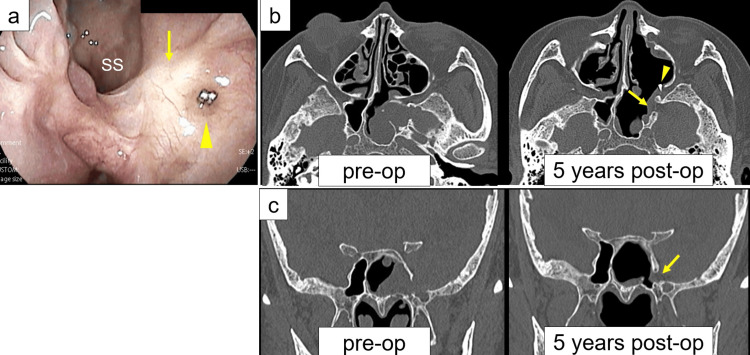
Findings at five-year follow-up after surgery. (a) Nasal endoscopy five years after surgery showing epithelialization of the cranial base reconstruction site, with vascular clips visible on the mucosa. (b) Axial HRCT views before (left; also shown in Figure 1b) and after (right) surgery, demonstrating reconstruction of the skull base defect with no recurrence. (c) Coronal HRCT views before (left; also shown in Figure 1c) and after (right) surgery, confirming stable reconstruction with no recurrence of the meningocele. Preoperative images are presented alongside postoperative images for direct comparison. The arrows on panels (a-c) indicate the reconstruction site. The arrowheads on panels (a) and (b) indicate vascular clips. SS, sphenoid sinus

The patient had no surgical complications, and the postoperative course was uneventful. There were no signs of CSF leakage or meningitis recurrence during the five years of follow-up. Nasal endoscopy (Figure 4a) and CT (Figures 4b, 4c) performed five years following surgery revealed complete mucosal epithelialization and no evidence of meningocele or residual skull base defect. These findings confirmed the durability of the multilayer closure and the success of the EETPA.

## Discussion

Epidemiology and anatomic predisposition to lateral sphenoid meningoceles

Meningoceles arising from the lateral recess of the sphenoid sinus are uncommon, accounting for a small subset of spontaneous CSF leaks [3]. The lateral recess is susceptible to such defects owing to extensive pneumatization and thin bony walls, particularly when the sinus extends beyond the VR line [1,8,9]. Anatomical studies have described substantial variability in pneumatization patterns, and this variability may predispose this region to spontaneous skull base defects [11,12].

Diagnostic lessons from the literature

Patients with lateral sphenoid meningoceles, similar to other causes of CSF leak, often present with nonspecific symptoms such as serous rhinorrhea or recurrent meningitis, as in the present case. The diagnostic workup should include HRCT to detect bony dehiscence and MRI to identify a meningocele or encephalocele. A positive β-2 transferrin test is critical for confirming CSF leakage. However, previous interventions, such as surgery for presumed sinusitis, can obscure the clinical picture and delay definitive diagnosis [1].

As highlighted by Shimizu et al. [9], delayed or incorrect diagnosis of sphenoid sinus meningocele is a common pitfall, particularly in patients who have undergone previous sinonasal surgery or who present without overt CSF rhinorrhea. Their report of a patient initially misdiagnosed with isolated sinusitis emphasizes the importance of maintaining a high index of suspicion. Our case also demonstrates that subtle imaging findings and non-specific intraoperative observations may obscure the true etiology unless lateral skull base defects are specifically considered.

Based on their institutional experience and literature reviews, several researchers [4-7] cite the presence of Sternberg’s canal as a predisposing factor for spontaneous meningocele. Their analyses support targeted imaging assessment of this region and strengthen the rationale for using EETPA in cases involving the lateral recess.

Surgical approach selection: rationale for EETPA

The EETPA has emerged as the preferred technique for accessing lesions located in the lateral recess of the sphenoid sinus - particularly those located lateral to the VR line [1,8,9]. Compared with traditional transnasal or transsphenoidal routes, EETPA provides enhanced visualization of critical anatomical structures, including the pterygopalatine fossa, foramen rotundum, and V2, allowing for safer and more effective exposure of lateral skull base defects [1].

In the present case, preoperative HRCT and MRI clearly demonstrated a bony defect lateral to the VR line, suggesting that a standard transsphenoidal approach would not provide sufficient access. EETPA enabled wide exposure of the lateral recess, facilitating complete visualization of the defect and safe dissection. A recent systematic review by Ahmed et al. comparing endoscopic endonasal and combined transorbital-endonasal approaches for nontraumatic lateral sphenoid recess CSF leaks reported a 95.2% repair success rate for the endoscopic endonasal approach, supporting its role as a primary strategy in appropriately selected cases [13]. The anatomical and radiologic findings, combined with the known advantages of EETPA in lateral skull base access, confirmed its suitability in this case.

While the EETPA was selected in this case due to the defect’s location lateral to the VR line, alternative approaches, such as the transorbital neuroendoscopic approach, have also been reported for lateral sphenoid recess CSF leaks. These may be considered in selected cases depending on defect orientation, surgeon expertise, and preservation of vascular structures.

Downstream implications of intraoperative SPA transection for reconstruction strategy

Transection of the SPA is sometimes necessary during the EETPA to gain sufficient access to the lateral recess [2]. In our case, transecting the SPA precluded the use of an ipsilateral NSF. Although the options for vascularized reconstruction were limited, the approach allowed for safe and complete resection of the meningocele.

Several studies [9,10] have emphasized the importance of careful preoperative planning regarding SPA preservation. Transection of the SPA may be required for exposure, particularly for lesions involving the lateral recess or pterygopalatine fossa, but sacrificing the vessel should be weighed against its potential role in supporting vascularized flap viability [9,10]. In our case, given the lateral location of the defect and the prior surgery, the benefit of broader exposure outweighed the loss of the SPA.

Reconstruction techniques and materials

Reconstructing lateral skull base defects requires a secure, multilayered closure. Autologous grafts such as fascia lata and fat are commonly used owing to their biocompatibility and accessibility [14]. In this case, the combination of thigh-derived fascia lata and fat, augmented with synthetic Neoveil® and fibrin glue, provided sufficient closure. Despite the lack of a vascularized flap, a watertight seal was achieved, obviating the need for additional flap harvest. Our reconstruction technique is supported in the literature by Hong, who emphasized that, when a vascularized flap is not available or viable because of prior interventions or vessel sacrifice, layered autologous grafting supplemented with synthetic materials may yield reliable outcomes [14].

We would also like to clarify that the skull base defect was reconstructed using a multilayered closure technique with autologous fascia lata, fat, and synthetic material, achieving watertight sealing confirmed by nasal endoscopy and CT at five years postoperatively. The absence of recurrence during long-term follow-up supports the durability of the repair.

Role of lumbar drainage in skull base repair

The use of lumbar drainage remains controversial. Whereas some advocate routine LD placement postoperatively to reduce intracranial pressure, the recent literature supports selective drain use based on intraoperative assessment [12]. In our patient, an LD was deemed unnecessary because of satisfactory reconstruction and the absence of postoperative CSF leakage. In a case similar to ours, Yıldırım et al. reported successful watertight closure, without using an NSF or LD, with no recurrence of CSF leakage [15]. Furthermore, Hannan et al. reported that iterative refinements to a standardized multilayer repair protocol for high-flow CSF leaks allowed achievement of a postoperative leak rate as low as 4% without nasal packing or lumbar drainage, reinforcing the safety of omitting these adjuncts when closure is robust [16]. However, an LD remains a useful adjunct in salvage settings, particularly when primary closure fails or CSF leakage persists postoperatively [12].

Long-term outcomes and follow-up considerations

Our patient’s postoperative recovery was uneventful. Nasal endoscopy and CT at five years postoperatively confirmed complete mucosal healing without recurrence of the meningocele or CSF leakage (Figure 4). Since the case report of recurrent CSF leakage more than one year after surgery [2], long-term follow-up with nasal endoscopy and imaging has been strongly recommended. Our case highlights the importance of individualizing reconstruction strategies tailored to intraoperative findings.

## Conclusions

This case highlights the clinical and surgical nuances that must be considered when managing spontaneous lateral sphenoid sinus meningoceles. The use of EETPA enabled successful exposure and resection of the lesion, despite the altered surgical field. Furthermore, despite intraoperative SPA ligation, multilayer reconstruction by using autologous fascia, fat, and synthetic materials proved effective in preventing CSF leakage.

Meticulous preoperative planning, intraoperative decision-making, and tailored reconstruction performed by a multidisciplinary team are all critical for achieving optimal outcomes in complex skull base defects.
